# Resistance to Fracture of Lithium Disilicate Feldspathic Restorations Manufactured Using a CAD/CAM System and Crystallized with Different Thermal Units and Programs

**DOI:** 10.3390/ma14123215

**Published:** 2021-06-10

**Authors:** Cristian Abad-Coronel, Andrea Ordoñez Balladares, Jorge I. Fajardo, Benjamín José Martín Biedma

**Affiliations:** 1 Faculty of Dentistry, Universidad de Cuenca, 010107 Cuenca, Ecuador; 2 Faculty of Dentistry, Universidad de Guayaquil, 090514 Guayaquil, Ecuador; andrea.ordonezb@ug.edu.ec; 3 Mechanical Enginnering Faculty, Universidad Politécnica Saleciana, 170517 Cuenca, Ecuador; jfajardo@ups.edu.ec; 4 Faculty of Dentistry, Universidad de Santiago de Compostela, 15782 Galicia, Spain; benjamin.martin@usc.es

**Keywords:** lithium disilicate, crystallization, fracture, CAD/CAM materials

## Abstract

The aim of this study was to determine the resistance to fracture of feldspathic restorations with lithium disilicate and crystallized with different ovens and programs. Methods: Sixty monolithic restorations (LD) (EMAX CAD™ LT, Ivoclar-Vivadent™) were designed with the same parameters and milled with a CAD/CAM system (CEREC SW 5.1, CEREC MCXL, Dentsply-Sirona™, Bensheim). Each restoration was randomly assigned by randomization software (RANDNUM) to one of the three groups: (a) (NF) Oven P310 (Ivoclar, Vivadent) normal crystallization program, (b) (FF) Ivoclar P310 oven (Ivoclar-Vivadent™) rapid crystallization program, or (c) (SF) SpeedFire oven (Dentsply-Sirona™). Results: There were statistically significant differences between the groups (ANOVA, *p* < 0.05). The NF and FF groups showed the highest values of resistance to fracture, with statistically significant differences with the SF group. Conclusions: Using a furnace from the same dental company with predetermined programs from the material manufacturer, as well as using a predetermined program for rapid crystallization, has no effect on fracture resistance, and would save clinical time when performing ceramic restorations with lithium disilicate, while keeping their mechanical properties.

## 1. Introduction

Ceramic CAD/CAM blocks were introduced to the dental market in 1980 [1]. Since then, they have gained great popularity in clinical practice, as the restorations made with this material are metal-free and allow the reconstruction of lost dental tissues with adequate mechanical and aesthetic properties [2]. Currently, there is a great demand for this type of restoration, based on the migration of conventional laboratory procedures to the recurrent use of digital technology, and due to the multiple advantages it offers [3], such as performing indirect restorations in the same session, with procedures including computer-aided design and manufacturing (CAD/CAM). Companies have developed various materials for CAD/CAM systems, incorporated in chairside systems, and intended for use in the dental clinic or in specialized prosthetic laboratories [4]. The characterization of CAD/CAM materials is a current trend in research, due to their predominant use in restoration protocols. One of the CAD/CAM materials most used for indirect restorations is feldspathic ceramic with lithium disilicate crystals, EMAX-CAD™ (Ivoclar-Vivadent™, Liechtenstein), which was developed in 2005, and which in its pre-crystallized or metasilicate intermediate state shows a purple color due to the stains used to identify this state. This material in the metasilicate state requires a certain time and temperature to reach its crystallized final state. The appropriate combination is usually between 800 °C and 870 °C, for 5 to 30 min. The mechanical properties of EMAX-CAD™, with a strength of 130–150 MPa, a fracture toughness of 0.9 to 1.25 MPa·m^1/2^, and a Vickers hardness of 5400 Mpa, are improved after receiving the definitive thermal treatment, at the same point that the material reaches its definitive tooth color [5,6,7,8].

Likewise, furnaces designed for post-milling crystallization heat treatment of the mentioned materials have been recommended. These ovens have been programmed with faster crystallization times, and the question remains if this will affect the final state of the restorations. Other manufacturers have launched thermal units with smaller firing chambers that also aim to improve the time used for post-treatment of materials machined in the precrystallized state; therefore, it is necessary to know if this treatment is adequate for achieving properties, such as resistance to fracture, equivalent to those obtained with other units. Furnaces from the same manufacturer’s catalogue (Programat™, P310, Ivoclar-Vivadent™, Liechtenstein) have been recommended for post-treatment of their materials [9]. Another furnace, SpeedFire™ (Dentsply-Sirona™, Bensheim, Germany) has been launched onto the market, the characteristic of which is determined by the CAM software and its connection with the thermal unit to determine the crystallization time of the restoration, depending on the material [10]. These ovens have programs, varying in time and temperature, which are important factors to consider for these CAD/CAM materials.

The crystallization process and the cooling rate are variables that influence the mechanical characteristics of this type of material; even an increase in the number of crystallization cycles can compromise the optical results [11,12,13]. The thicknesses of crystalline-reinforced ceramic restorations can also influence the esthetic appearance after repeated firings. The thinner the restoration, the greater the probability of changes with respect to its translucency [13]. Other characteristics, such as the marginal gap, can be modified due to the crystallization of the ceramic material with lithium disilicate. When quantifying the effect of the crystallization process of lithium disilicate ceramic crowns using CAD/CAM technology, comparing the values of the gaps in the marginal area and the internal areas of each crown showed that the former were greater and the latter were smaller after the crystallization process. The crystallization process can have a greater influence than the milling process itself on the variables studied, such as the marginal gap and the comparison between different CAD/CAM materials. It has been determined that it is the repeated crystallization firing process that plays the main role; giving a significant increase in the marginal space of lithium disilicate ceramics compared to feldspathic ceramics reinforced with leucite, probably due to the additional heating that causes a shrinkage of the ceramic in the margin [14,15,16].

Chairside systems aim to optimize clinical time and complete restorations in the same visit. To make restorations, the time taken is an important variable to consider. The less time taken without affecting the properties of the CAD/CAM materials, the more efficient the process will become [17,18].

On the other hand, both resistance to fracture and thermocycling are tests that allow knowing the behavior that restorations would have in the oral environment, so their use in in vitro studies allows a greater similarity with clinical scenarios [19,20].

The null hypothesis raised was that there would not be significant differences in the fracture resistance of lithium disilicate feldspathic CAD/CAM restorations subjected to different heat treatment units and programs for crystallization. The importance of this study lies in the fact that the materials and equipment used in these manufacturing technologies, focused on the area of prosthodontics, require both clinical and laboratory investigations in order to make adequate decisions about their use without affecting their operation and precision.

## 2. Materials and Methods

Sixty single upper first molar feldspathic CAD/CAM reinforced with lithium disilicate crowns on abutment replicas with three different thermal treatments were used in this study (Table 1).

### 2.1. Crown Design

**Figure 1:**
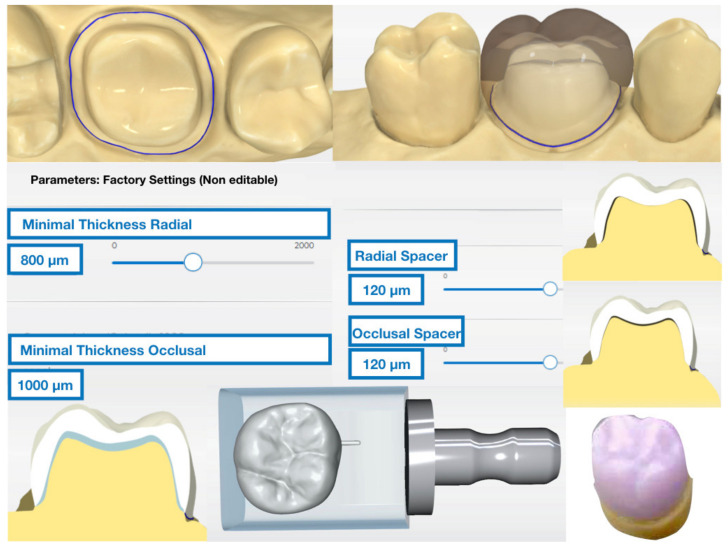
Demarcation of the chamfer finish line of the digital model, design of the crown with specific parameters for the spacer, minimal thickness and milling of the restoration.

A model of an upper molar prepared for full crown with a chamfer finish line was scanned using a high-power structured light scanner (PrimeScan™, Dentsply-Sirona™, Bensheim, Germany). After the model was digitized, a 1.25-mm thick full volume restoration was designed with integrated design software (CEREC SW 5.1, Dentsply-Sirona™, Bensheim, Germany). The parameters “minimal thickness (radial)” and “minimal thickness (occlusal)” were adjusted to 1000 μm. Other than that, the standard design parameters recommended by the manufacturer were used for the crown (Figure 1). Twenty monolithic CAD/CAM restorations made of lithium disilicate-reinforced feldspathic ceramic (EMAX-CAD™ LT, A2, LOT Z016FG, Ivoclar-Vivadent™; Schaan, Liechtenstein) per study group were milled using an integrated milling machine (CEREC InLab MCXL™ system, York, PA, USA).

### 2.2. Fabrication of CAD/CAM Abutments and Restorations

**Figure 2:**
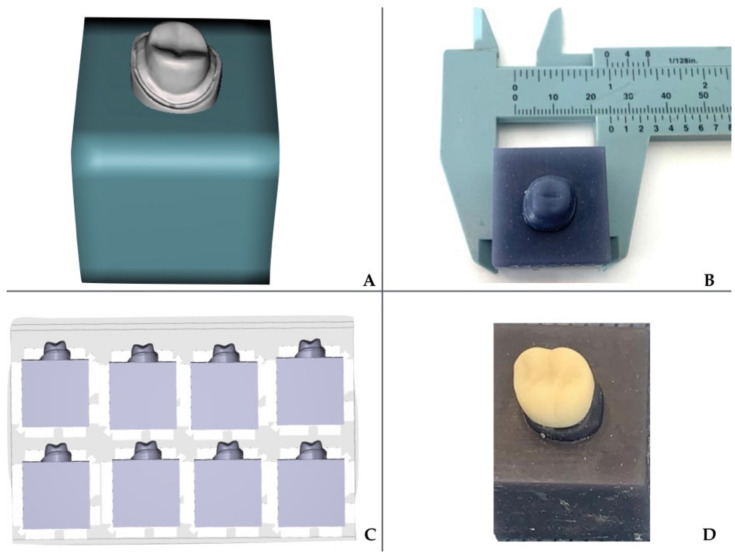
Digital file of the abutment exported from the scanned model. (**A**) Digital design of the block to adapt the abutment to the universal testing machine. (**B**,**C**) A cube of 25 mm^3^ was printed with the 3D printer for each crown. (**D**) Final restoration seated to the resin-printed die, previous to the loading test.

The die model was exported as a STL file to universal software (MESHMIXER™ 3.5, Autodesk, San Francisco, CA, USA). The abutment was digitally integrated into a 25 mm x side square specimen, to adapt it to the testing machine. The design was printed using a 3D printer with DLP technology (SprintRay™, Pro, Los Angeles, CA, USA) with layers of 50 microns definition, with a resin for models (Sprint Ray™, Gray Die, and Model, Los Angeles, CA, USA) (Figure 2).

### 2.3. Thermocycling of the Restorations

A thermocycler (Thermocycler™, SD Mechatronik, Feldkirchen-Westerham, Germany) with distilled water baths of 5 and 55 °C was used. The samples were aged for 5000 thermocycles in distilled water. Samples were subjected to 5000 cycles to estimate 5 years of oral conditions.

### 2.4. Crystallization Process of the Restorations

Once milled, the restorations underwent the crystallization process. Each restoration was randomly assigned using randomization software (0.1.1, RANDNUM™, San Francisco, CA, USA) to one of the three study groups (Table 1).

### 2.5. Fracture Loading of the Restorations

**Figure 3:**
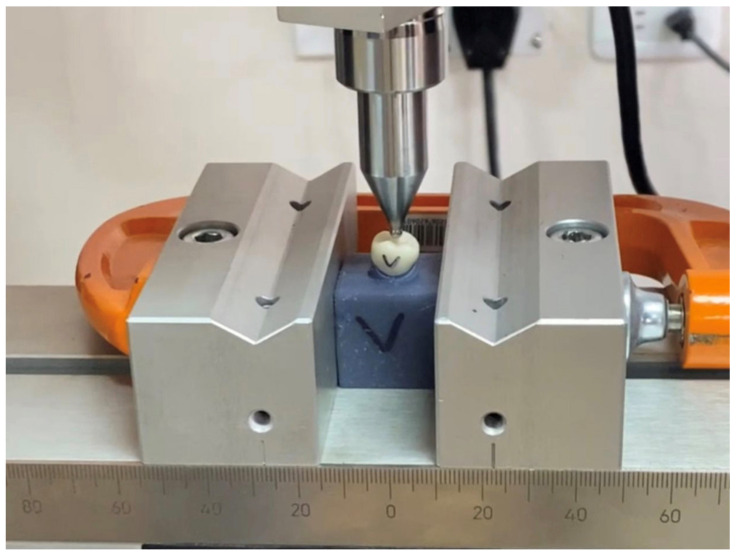
Loading of the specimen seated over the printed resin abutment using a universal testing machine.

Each restoration was placed on the respective resin die in the correct position. Each sample was subjected to a static load test at a speed of 0.5 mm/min with a direction parallel to the major axis of the tooth, and with an initial preload of 10N using a universal testing machine (Test Resources™, Series 300, Shakopee, MN, USA) equipped with a 5 kN load cell. The load was applied with a tempered steel pilot punch with a radius of 3 mm applied in the central fossa of the restoration. All specimens were loaded until fracture, and recorded in Newtons (N) by a computer connected to the testing machine (Figure 3).

### 2.6. Statistical Analysis

The data were collected in an EXCEL™ (Microsoft, Redmond, WA, USA) data sheet for descriptive statistical analysis calculating the mean, minimum, and maximum values, and standard deviation. (SPSS™ v. 21.0 software, SPSS Inc., Chicago, IL, USA). Fracture resistance outcomes were normally distributed. One-way ANOVA test was used to compare mean fracture resistance between the three study groups (NF, FF, SF), followed by reporting of simple main effects for each group. Bonferroni adjusted post hoc tests were used to assess differences between the three groups. The significance level was established at 5% (*p* < 0.05).

## 3. Results

**Figure 4:**
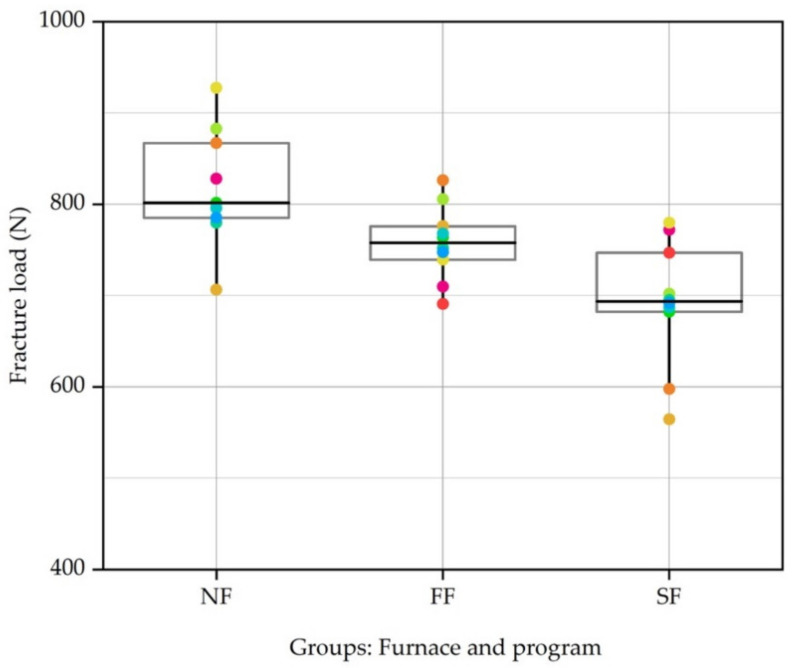
Fracture load (N) according to different thermal units and programs used.

The one-way ANOVA test revealed significant differences between the groups (*p* < 0.05), without specifying which of these three groups presented differences between them (*p* < 0.001, F = 8.699) (Table 2). After that, the Bonferroni post hoc test, showed that, in particular, the SF group exhibited statistically lower fracture resistance than the NF group (*p* < 0.001) and the FF group (*p* = 0.025) (Table 3). However, a statistically significant difference was not found between the NF and FF groups (*p* = 0.537) (Figure 4).

## 4. Discussion

This research aimed to compare, through an in vitro study, the resistance to fracture of feldspathic crowns reinforced with lithium disilicate and crystallized using two different thermal units and the three respective programs for the effect. The null hypothesis that the fracture resistance in the three groups of different thermal units and their respective specific programs for the crystallization of lithium disilicate materials would not present differences was rejected (Table 2). When performing restorations using a CAI (computer aided instruction) workflow and using softer materials for milling, they need to be crystallized, thus, increasing the time required for completion and finishing. It is known that the crystallization of materials causes changes in their microstructure, improving their mechanical properties [12,21]; since the crystallization firing cures the microcracks generated in the material by the grinding process. The ratio of glassy composition to crystalline phase changes from milling to crystallization firing, in addition to the transformation of a weak pre-crystallized metasilicate, which measures in a range from 0.2 µm to 1 µm, into stronger lithium disilicate crystals, which measure from 0.5 µm to 5 µm post-process; This determines the final state of the restoration with respect to its mechanical properties [22,23,24]. For chairside clinical protocols, time can be a key factor in obtaining definitive restorations, while keeping certain physical properties intact, such as resistance to fracture. According to the results of this study, using programs with reduced time does not affect the mechanical resistance of the restorations when compared with the results obtained with the same thermal unit but with a program that takes more time.

On the other hand, using different thermal units than those recommended by the manufacturer slightly affects the final resistance of the restorations. It has been established that adjusting some crystallization parameters can increase the reliability of the properties of the restorations. In the rapid crystallization group (FF), the final temperature did not exceed 870 degrees, with a faster rate of rise, finishing the process in 15 min with faster cooling. This was the group where the resistance of the restorations was slightly lower than those obtained in the crystallization group with a specific program from the same manufacturer, and where the time to finish the process was longer (almost 24 min), but with a final temperature of 850 degrees and without statistically significant differences after the post hoc test comparison between groups. In SpeedFire, a time of 24 min was determined automatically by the oven connection software at a final temperature of 797 degrees for the same standardized restoration. This takes into account that, in certain cases, SpeedFire can add more firing time depending on the size and thickness of the restoration. In this group (SF), the fracture resistance values obtained were lower, with statistically significant differences with the other two groups that used a furnace with the manufacturer’s specific programs (Table 3). The lower final temperature could have influenced the final resistance to fracture of the crystallized restorations in this furnace. The thicknesses of all restorations were identical; therefore, it was important to standardize the samples to evaluate the behavior of the material, which can be achieved through a digital workflow. According to our study and previous research, a final temperature value between 830 and 870 degrees allows an effective crystallization process to be achieved, while managing to mitigate the effects of the milling processes [11,25]. These temperatures were only achieved with specific programs of the oven and material manufacturer. Within the mechanical properties of the materials, the evaluation of the resistance to fracture is a decisive step in predicting the behavior of the restorations prior to the loads to which they are subjected within the masticatory function. Ceramic materials have been shown to withstand loads that exceed the values during this function [26]. In this study, the resistance values of the three groups of crowns were within the established ranges required in international standards for single restorations, agreeing with the statement that they can be used even in posterior sectors [27,28]. Likewise, with these antecedents, the clinical success of restorations made with this material could be substantiated [29]. Specimens in the form of anatomical crowns with a thickness of at least 1.84 mm, could withstand the initial load of the cell used, depending on an adaptation of tests used to measure fracture toughness, and this could be recommended as the ideal thickness [30]. Although one study suggested that even a thickness of 1 mm could be sufficient to achieve durability for these types of restoration [31], a recommended intermediate thickness of 1.25 mm was used for the occlusal aspect of the restorations in this study. Designing and using these test specimens in vitro, as close as possible to the ideal scenario, would allow a better prospective evaluation of what could happen in clinical situations under compressive loads. The values obtained in this study are in agreement with the minimal results of axial loads on restorations made with the same material (788 N) [31,32].

The digital flow allows the standardization of all samples from both crowns and dies. The fracture resistance values of ceramic materials with lithium disilicate reinforcement can be affected depending on the substrate [33]. Considering this, the dies were designed in a standardized way and were resin-printed to approximate the elastic modulus of dentin and to simulate the clinical setting where restorations are performed. The restorations were not cemented, so the resistance values are exclusively attributable to lithium disilicate ceramics; therefore, the values may be lower than in other studies where the crowns were cemented. Crystallization is a process that generates changes in the structure of the material, it has even been reported that it can cause changes in the marginal adjustment [14], although these changes in the marginal zone are below the clinically accepted threshold [15]; therefore, it is important to recommend more investigations to discern the variables of cooking times and final temperature, with respect to the marginal adjustment.

The limitations of this study were not presenting a fractographic analysis to determine the beginning and trajectory of the cracks caused by the load. Isolating the variable of adhesive cementation that is generally used clinically to bond restorations, and with which the resistance to fracture is diminished in this study, has also previously been attempted. It would also be useful to establish the influence of the vacuum on the thermal processes applied in the different furnaces.

## 5. Conclusions

It should be emphasized that within the limitations of this in vitro study, in trying to isolate variations inherent to the oral environment and looking for changes only by analyzing the variables related to the thermal units, it was established that the specific programs of the manufacturer for the thermal units of crystallization and lithium disilicate ceramic material showed time optimizations without affecting the mechanical properties of the lithium disilicate ceramic restorations. The fracture resistance values considering the manufacturer’s recommendations showed significant differences between the fracture resistances of the three study groups. The results of this study encourage a clinical study evaluating the longevity of lithium disilicate ceramic restorations. More studies on the mechanical properties and different crystallization programs, times, and temperatures are recommended.

## Figures and Tables

**Table 1:** Furnaces, groups, and program specifications used in this study.

Furnace	Manufacturer	Crystallization Programs
Programat P310	Ivoclar-Vivadent™/Liechtenstein	**Group A, Normal Firing (NF)** final temperature 850 °C/approximately 23 min 50 s **Group B, Fast Firing (FF)** final temperature of 870 °C/15 min 10 s
Speed Fire	Dentsply-Sirona™/Bensheim, Germany	**Group C, SpeedFire (SF)** final temperature 797 °C/approximately 24 min

**Table 2:** One-way ANOVA factor for the fracture resistance.

Groups	Sum Square	Gl	Mean Square	F	Sig.
Inter-groups	101077.700	2	50538.85	8.699	0.001
Intra-groups	331141.965	57	5809.50	-	-
Total	432219.665	59	-	-	-

**Table 3:** Bonferroni post hoc test. *Significative differences were found.

Furnace Type	Group	Mean Difference (I–J)	Sig.	C. I. 95%	
				Lower Limit	Upper Limit
Normal Firing	Fast Firing	32.80	0.537	−26.64	92.25
	Speed Fire	98.70 *	0.000	39.25	158.15
Fast Firing	Normal Firing	−32.80	0.537	−92.25	26.64
	Speed Fire	65.90 *	0.025	6.44	125.35
Speed Fire	Normal Firing	−98.70 *	0.000	−158.15	−39.25
	Fast Firing	−65.90 *	0.025	−125.35	−6.44

## Data Availability

Data is contained within the article.
